# Phenotypic alterations in human saphenous vein culture induced by tumor necrosis factor-alpha and lipoproteins: a preliminary development of an initial atherosclerotic plaque model

**DOI:** 10.1186/1476-511X-12-132

**Published:** 2013-09-08

**Authors:** Kriengchai Prasongsukarn, Urai Chaisri, Peenutchanee Chartburus, Kamolwan Wetchabut, Surachet Benjathummarak, Vasant Khachansaksumet, Yaowapa Maneerat

**Affiliations:** 1 Pramongkutklao Hospital, Bangkok, Thailand; 2 Department of Tropical Pathology, Faculty of Tropical Medicine, Mahidol University, 420/6 Ratchawithee Rd, BKK, Bangkok, 10400, Thailand; 3 The Institute of Cardiovascular Diseases, Rajavithi Hospital, Bangkok, Thailand; 4 Center of Excellence for Antibody Research, Faculty of Tropical Medicine, Mahidol University, Bangkok, Thailand

**Keywords:** TNF-alpha, LDL, Atherosclerosis, Saphenous vein

## Abstract

**Background:**

Atherosclerosis is a chronic progressive inflammatory disease of blood vessels particularly the arteries. The development of atherosclerotic plaques or atherogenesis is a complex process that is influenced by cardiovascular risk factors such as vascular inflammation and dyslipidemia. This study demonstrates the ability of tumor necrosis factor-alpha (TNF-α) and low density lipoproteins (LDL) to induce atherosclerotic plaque in human saphenous vein (HSV) organ culture.

**Methods:**

Normal HSV segments, from male patients who had coronary bypass graft, were cultured in DMEM containing 5% heat inactivated fetal bovine serum. TNF-α (5 ng/ml) was applied in combination with native LDL (nLDL) or oxidized LDL (oxLDL) at the dose of 50 μg/ml for 14 days. The phenotypic changes of the organ cultures characteristic of initial atherosclerotic plaques were evaluated. The effect of anti-atherogenic agent, 17-β estradiol (E_2_), was also determined.

**Results:**

Histologic, histomorphometric, and immunohistochemical examinations revealed that HSV rings stimulated with TNF-α + nLDL or TNF-α + oxLDL can exhibit the essential morphological features of atherogenesis, including fibrous cap formation, cholesterol clefts, evident thickening of the intimal layer, increased proliferation of smooth muscle cells (SMC) and migration to the subendothelial layer, significant SMC foam cell formation, and increased expression of adhesion molecules in the vascular wall. Addition of E_2_ (50 nM) to the culture significantly modulated the critical changes. Consistently, mRNA profiling of the HSV model revealed that 50 of 84 genes of atherosclerosis were up-regulated.

**Conclusions:**

Phenotypic changes characteristic of the initial development of atherosclerotic plaques can be induced in HSV organ culture.

## Background

Atherosclerosis, one of the most important vascular diseases worldwide, is a complex and progressive disease characterized by accumulation of lipids and fibrous elements in large arteries [1]. Epidemiological studies have revealed numerous risk factors for atherogenesis, including gender, smoking, hyperlipidemia, inflammation, and genetic background [1]. Histopathology studies of the vascular changes during atherogenesis have shown that blood derived inflammatory cells, particularly monocyte/macrophages, play a key role in the initiation and progression of the disease. Highly inflammatory monocytes express increased levels of immunogenic factors such as Toll-like receptors (TLR) and cytokines, e.g., TNF-α and IL-1 [2]. TNF-α is an important pro-inflammatory cytokine which is involved in endothelial cell (EC) dysfunction and atherosclerosis by down-regulating endothelial nitric oxide synthase (eNOS) expression [3]. A decrease of endothelial nitric oxide production synthesized by eNOS causes several critical features of vascular inflammation associated with atherosclerosis. These include increased adhesion molecules on EC, elevated platelet aggregation and adhesion on EC, enhanced inflammatory cell binding to EC and recruitment to the subendothelial layer, and smooth muscle cell (SMC) proliferation and migration [3]. Consistently, activation of TNF-α was demonstrated in human atherosclerotic plaques and proliferating SMCs in the balloon-injured rabbit aorta [4].

LDL is another important key factor of atherogenesis as evidenced by the correlated incidence of hyperlipidemia atherosclerosis in human patients [1], and various animal models including pigs, rabbits, rats, and mice [5]. Earlier studies reported that oxidation of LDL has important biological effects in atherosclerotic plaque development, particularly in foam cell formation by macrophage and SMC [5,6]. Vascular endothelial dysfunction at the initiation phase of atherogenesis is also correlated with the introduction of extracellular lipid into the T. intima (TI) of the arterial wall. In the lipid accumulation phase, monocytes that has migrated into the arterial wall differentiate into macrophages and arise to express scavenger receptors (ScR) that bind, and facilitate the uptake of modified lipoproteins. Over time, monocyte-derived macrophages develop into macrophage foam cells. Macrophage foam cells and other cells in the vascular wall produce various cytokines and growth factors that promote SMC migration and proliferation [2].

Nowadays it is necessary to gain better understanding of the pathogenesis of human atherosclerosis and relevant genetic and pharmacological factors. The developments of atherosclerosis have been widely studied in a variety of large and small animal models such as rabbits, rats [5], pigs [7], non-human primates [8], and most commonly in mice [reviewed in 5], but human models are rare. Most *ex vivo* and *in vitro* studies in human atherosclerosis and other vascular diseases have been performed with organ (vessel) culture techniques [9-11], and co-culture of endothelial cells and SMC from umbilical [12], HSV [13] or the aorta [14]. In advantage, the organ culture techniques preserve the *in vivo* anatomic relationships such as the vascular cell organization in the extracellular matrix which control the vascular response to injury [11].

Our previous atherosclerosis-related model was demonstrated in TNF-α stimulated co-culture of EC and SMC obtained from umbilical veins [12]. The atherosclerotic criteria by TNF-α induction, including increased adhesion molecule expression and platelet aggregation were principally caused by EC and eNOS dysfunction [9,12]. However, this atherosclerotic model [12] has noticeable limitations such as the lack of neointima hyperplasia, foam cell formation, and SMC proliferation and migration to the TI of the vascular wall.

To improve our previous *in vitro* model, this study develops a simple initial atherosclerosis plaque model using HSV organ culture induced with a combination of TNF-α and nLDL (T + nLDL) or oxLDL (T + oxLDL). Our results revealed phenotypic changes that were consistent with the atherosclerotic plaque features. The principle underlying our current approach was the theory of “Response to Injury” [5]. Firstly, TNF-α induces dysfunction of EC [2,12] and expression of ScR on EC and SMC [15,16]. Subsequently, oxLDL or oxidized nLDL in the culture media mimics hyperlipidemia status in patients [5] and passes through EC lining and TI via ScR [16]. Our new model here can produce several phenotypic hallmarks of atherogenesis. Importantly, many of these are susceptible to anti-atherosclerotic agent 17-β estradiol (E_2_).

## Methods

### Materials

In culture technique, we used Dubecco’s Modified Eagle’s Medium (DMEM), fetal bovine serum (FBS) (Gibco), TNF-α, E_2_ (Sigma-Aldrich, St. Louis, MO). nLDL and oxLDL were from Kalen Laboratories. Antibodies to VCAM-1 (Santa Cruz biotechnology, Inc, California, USA), alpha actin (Dako, Glostrup, Denmark), biotinylated anti-PCNA (Biolegend, San Diego, CA, USA), strepavidin–peroxidase kit and peroxidase substrates (Vector laboratories, Burlingame, CA, USA) and Weigert staining kit (Bio-Opica, Milan, Italy) were used in immunohistochemistry and histomorphometry. Oil red O dye was from Sigma (St. Louis, MO, USA). Atherosclerosis DNA microarray kit and RNA extract kit were purchased from QIAGEN (Hilden, Germany). Reagents for electron microscopic study were from Electron Microscopy Sciences (Nashville, Tennessee, USA). The others were from Sigma-Aldrich.

### Subjects

Normal HSV segments, determined by routine histological inspection, were obtained from male patients undergoing coronary artery bypass graft surgery at Pramongkutklao Hospital and Rajavithi Hospital, Bangkok, Thailand. The donors had no clinical history of all infectious diseases. The ethical committee of Faculty of Tropical Medicine, Mahidol University, Pramongkutklao Hospitol and Rajavithi Hospital approved this study. All donors involved in this study were informed the objectives of this study and filled the consent forms. The HSV segment from each donor was conducted for an independent experiment.

### Experimental design

#### Vessel preparation and culture

Fresh HSV segments obtained from the hospital were transferred to the culture laboratory (YM) in cool medium, then immediately dissected connective tissue and adventitia. The vessels were divided into 3 mm length rings. The ring segments were quiescent overnight in DMEM supplemented with 1% of heat inactivated FBS before the experiments. Each HSV ring was cultured individually in 24 well plates with 1 ml of complete medium. The conditions in each independent experiment included HSV ring at D0, cultured in medium alone, medium with TNF-α, T + nLDL with/without E_2_ and/or T + oxLDL with/without E_2_ in duplicate.

#### Stimulation of atherosclerosis plaque like lesions in static condition

After overnight quiescence, the HSV rings were changed to culture in DMEM supplementd with 5% heat inactivated FBS [17], 5 ng/ml of TNF-α [12] plus 50 μg/ml of nLDL (T + nLDL) or oxLDL [18] (T + oxLDL) with or without E_2_ (25 - 50 nM). The 500 μl of medium was changed with fresh prepared medium with indicated conditions every two days until day 12.

#### Evaluation of feasibility of atherosclerosis plaque model

##### Determination of histopathologic changes by histopathology, histomorphometry, Immunohistochemistry technique

After 14 days of cultivation, the HSV rings were harvested to investigate morphology and histopathologic changes by standard hematoxylin & eosin (H&E) staining. The atherosclerotic plaques like lesions were examined by histomorphometric analysis (Verhoeff’s staining) [10], Masson trichrome staining [19], and immunostaining [12,20]. The foam cell formation in SMC was confirmed by Oil red O staining [21], and immunostaining of serial sections, and transmission electron microscopy (TEM) [12,22]. The effect of E_2_ to reduce critical features of atherosclerotic plaques was evaluated comparing among syngeneic HSV rings on Day 0, and those cultured in all conditions.

##### Modulation of atherosclerosis plaque like lesions

Atherosclerotic plaques like lesion in the HSV rings induced by T + nLDL or T + oxLDL in DMEM + 5% heated FBS [17] were modulated by adding E_2_ (25-50 nM) in the culture medium. After 14 days, the effect of E_2_ to modulate the critical features was evaluated comparing among HSV rings on day 0, and those cultured in all conditions.

### Tissue preparation

After cultivation, HSV segments were harvested, washed in PBS and fixed in 1% paraformaldehyde overnight at 4°C. Then each fixed segment was washed in 0.01 M PBS, crossly divided into two parts; one part was processed parafinnized block for histology, histomorphometry, and immunohistochemical studies [9,10]. Another part was processed for cryosection in 10 μm thick for lipid staining with oil red O method [21].

### Histology and histomorphometry

Serial consecutive paraffinized sections (4 μm) of HSV were deparafinized and rehydrated in serial baths of OTTIX, OTTIX Shapper, ethanol and PBS, then routinely stained with hematoxylin & eosin for histological examination. Histomorphometric analysis of thickness of TI and T. media (TM) were determined using Weigert staining kit under light microscope (Olympus; BX41). Masson’s trichrome staining was used to observe the extracellular matrix expansions into TI and TM [10].

### Immunohistochemistry 

After antigen retrieval for 10 min by boiling in 0.01 M sodium citrate, deparaffinized sections were quenched in 0.3% hydrogen peroxide for 30 min and incubated in 1% BSA in PBS for 30 min. Sections were incubated with primary antibodies: Rabbit anti-human VCAM-1 (1:200), alpha actin (1:1000) to identify SMC, or vWf (1:1000) to identify EC at room temperature for 45 min and then 4°C overnight. Samples were detected with secondary antibodies, biotinylated goat anti-rabbit or mouse IgG (Vector Laboratories, USA), for 30 min, washed, followed by streptavidin peroxidase for 30 min, and color visualized using 3, 3'- diaminobenzidine (DAB) for brown color, or Vector Red for brown-red color (Vector Laboratories, Burlingame, CA, USA). Finally, the sections were treated with Mayer’s hematoxylin for 1 min to stain nuclei. The negative control staining was omission of the primary antibodies. The positive control for PCNA staining was performed on human tonsillitis sections.

### Determination of PCNA expressed SMC

At least total 500 SMCs were examined at high power field in all specimens. The HSV sections were independently, and blindly counted by two observers (YM and UC). A proliferative index, defined as the numbers of positive nuclear staining SMC were represented in the percentage of total 500 SMCs [23].

### Semi-quantitative determination of adhesion molecule expression in vascular cells

Slides were investigated under a light microscope by two independent observers (YM and KW). Immunoreactivity was classified by estimating the percentage (P) of 300 total identified cells showing characteristic staining (from an undetectable level or 0%, to homogeneous staining or 100%) and by estimating the intensity (I) of staining (0, negative staining; 1, weak staining; 2, moderate staining; and 3, strong staining). The semi-quantitative expression results (S) were scored by multiplying the percentage of positive cells (P) of total by the intensity (I); S = P × I as modified from a previous study [24].

### Identification of foam cells by Oil red O staining

After air-dried, 10 μm thick HSV cryosections were soaked in DW for 5 min. The sections were then pretreated with absolute 2-propanol for 2 min and stained with 0.5% oil red O in 2-propanol at room temperature overnight. The slides were differentiated with 80% of 2-propanol, and then with DW. Finally, the cells were counterstained with Mayer’s hematoxylin for 1 min to localize nuclei. The slides were examined under light microscope. The intracellular lipid bodies were stained in red. Foam cells were defined as having ≥10 Oil Red O-positive lipid bodies per cell. Randomly 5 fields each from at least 3 sections were counted for the calculation [21].

### Ultrastructural investigation of foam cells by TEM

After cultivation, some HSV rings induced with T + nLDL or T + oxLDL were harvested and processed for TEM [12,22] to identify the lipid bodies in SMC foam cells. Briefly, harvested HSV rings were washed in 0.1M phosphate buffer (PB) with sucrose pH 7.2, fixed with 2.5% glutaraldehyde in PB at 4°C overnight. After post fixation in 1% osmium tetroxide in PB for 2 hours, the tissues were gradually dehydrated in acetone and embedded in epon-aradite. 70 nm ultrathin sections were prepared, double stained with uranyl acetate and lead citrate, and examined in a transmission electron microscope (Joel 1200 EX).

### Statistic analysis

All data are expressed as means ± standard error of mean (SEM). The data including the proliferative index, thickness of TI, number of foam cells, and scores of VCAM-1 expression were statistically analyzed among the groups using the Kruskal-Wallis test and the Mann-Whitney U test using SPSS 11.5 (Chicago, USA). A value of p < 0.05 was considered statistically significant.

## Results

### HSV stimulated with T + nLDL and T + oxLDL could induce atherosclerotic plaque liked lesions within 14 days

#### T + nLDL and T + oxLDL induced thickening of TI in HSV rings

**Figure 1:**
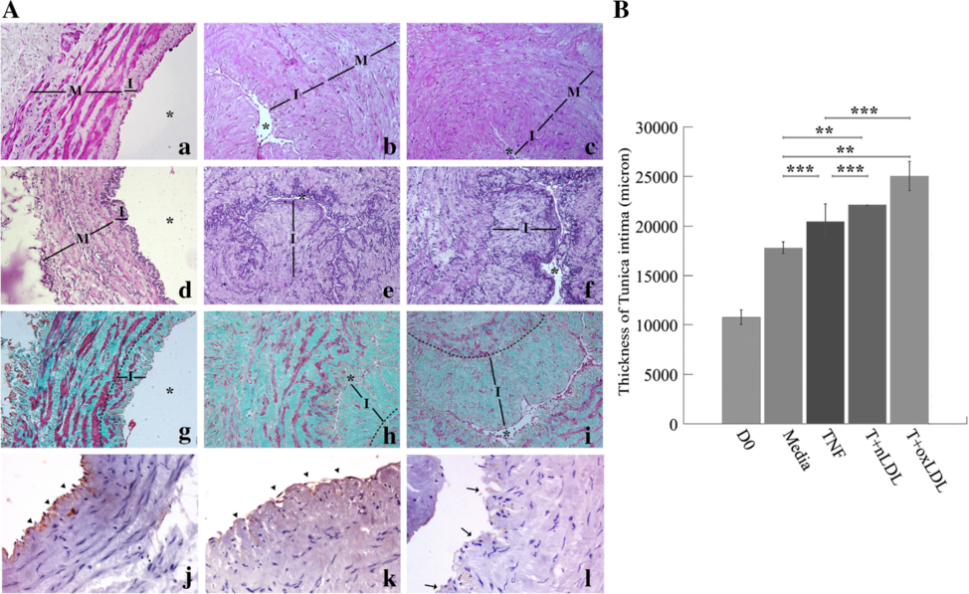
**T + LDL induced atherosclerotic plaque like lesion. (A)** Photomicrograph of HSV rings (original magnification x 200); harvested on day 0 **(a, d, g)**, and day 14 after cultured with T (5 ng/ml) + nLDL (50 μg/ml) **(b, e, h)** or T (5 ng/ml) + oxLDL (50 μg/ml) **(c, f, i)**. Histopathologic changes observed by H&E staining **(a, b, c)**, thickening of TI and expansion of extracellular matrix in vascular wall determined by Weigert staining **(d, e, f)** and Masson Trichrome staining **(g, h, i)**, respectively. * points lumen of HSV ring, I and M localize TI and TM, respectively. The endothelial lining of HSV rings at D0, cultured with TNF-α (5 ng/ml) or T + oxLDL **(j, k, l)** was localized by immunostaining with anti-VonWillebrand factor (vWf) and peroxidase substrate DAB (original magnification x 400). Arrowheads and arrows point the positive and negative reactivity of EC, respectively. The findings were obtained from one of similar six independent experiments. **(B)** Graph of thickening of TI compared among HSV rings cultured in medium alone, with TNF-α, T + nLDL, or T + oxLDL to the syngeneic HSV on day 0. The data represent mean ± SEM of T. intimal thickness (μm) of those cultured in medium alone, (n = 6). Significant differences among individual groups are indicated; ** p- valves < 0.05, and *** = not significant.

Our findings revealed marked histopathologic changes in 14-day T + nLDL or T + oxLDL stimulated HSV rings, including fibrous cap formation, thickening of TI, expansion of extracellular matrix in the TI and proliferation of SMC (Figure 1A). Immuno-localization of von Willebrand factor (vWF) in EC of HSV suggested that TNF-α alone could damage endothelial integrity, while T + oxLDL caused complete injury compared to the untreated HSV on day 0 (Figure 1A: j-l). The thickened vascular walls were composed of 1) SMC proliferation and migration to the subendothelial layer, and 2) expansion of extracellular matrix to the subendothelial layer in comparison to HSV rings on day 0 and cultured in medium (Figure 1). By examination of Masson Trichrome stained HSV sections, TNF-α alone, T + nLDL or T + oxLDL noticeably enhance thickening of the intimal layer in HSV rings comparing to the HSV on D0, and those cultured in medium (Figure 1B). The TI of HSV stimulated with T + nLDL (22,126 ± 126 μm), and with T + oxLDL (25,031 ± 2891 μm) were significantly thicker than those in the medium alone (17,788 ± 1,064 μm), p < 0.05. There was no significant difference in thickness of TI between cultured with TNF-α (20,444 ± 3,489 μm) and in medium (p = 0.434). The thickening of TI of HSV stimulated by TNF-α alone, T + nLDL (p = 1.000) or T + oxLDL (p = 0.355) were also not significantly different.

#### Proliferation of SMC in both TI and TM was indicated by significantly increased PCNA expression in SMC nuclei

**Figure 2:**
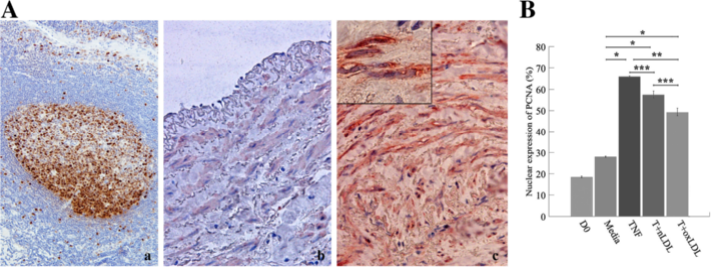
**Proliferation of SMC in HSV cultured with T + LDL. (A)** Nuclear expression of proliferating cell nuclear antigen (PCNA) by immunohistochemical staining; **(a)**, markedly localization of PCNA in nuclei of lymphoblasts in marginal zone of human tonsillitis section (PCNA positive control staining, original magnification x 200); b and c, double immunostaining of HSV rings (original magnification x 400); on day 0 **(b)** and day 14 after cultured with T (5 ng/ml) + oxLDL (50 μg/ml) **(c)**, PCNA in nuclei and alpha actin in cytoplasm of SMC, inlet with x1000 magnification. PCNA and alpha actin positive reactivity was visualized with peroxidase substrate DAB (brown color) and Nova Red (red color), respectively. **(B)** Percentages of PCNA expressed SMC in HSV rings were compared among all groups as mentioned above. Data are means ± SEM from 3 - 6 independent experiments. Significant differences among groups are indicated; * p- valves = 0.00, ** p- valves < 0.05, and *** = not significant.

Immunohistochemistry for alpha-actin (a marker of SMC) and PCNA in SMC in both TI, and TM represented the proliferation of SMC (Figure 2A). The comparison of proliferative index (percentages of PCNA expressed SMC) among groups was shown in Figure 2B. TNF-α (66.0 ± 0.38%, p = 0.000), T + nLDL (57.40 ± 3.19%, p = 0.000), and T + oxLDL (49.18 ± 3.43%, p = 0.001) treatments significantly promoted SMC proliferation. There was no significant difference in SMC proliferation between T + nLDL and T + oxLDL (p = 0.413), or TNF-α and T + nLDL induction (p = 0.563), or HSV at D0 and in the medium alone (P = 0.182).

#### SMC foam cell formation

**Figure 3:**
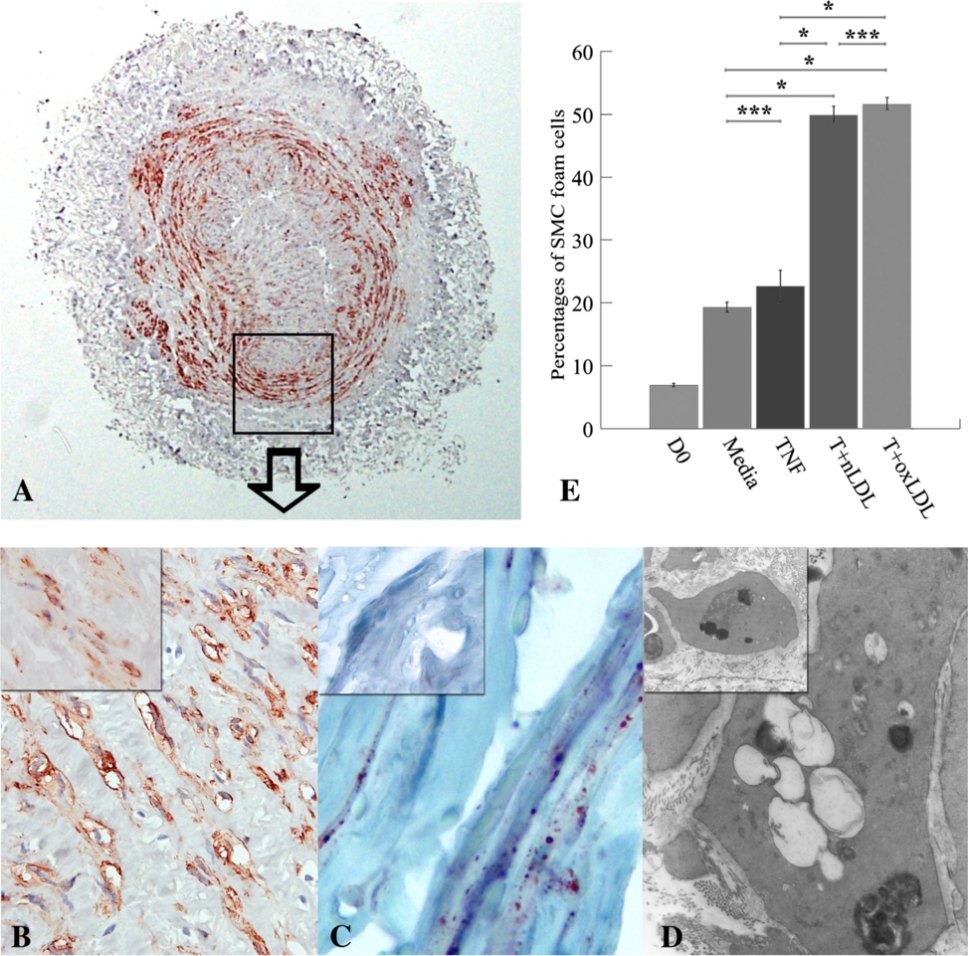
**Foam cell formation in HSV cultured with T + LDL.** The photomicrographs of SMC foam cells in a HSV ring stimulated with T (5 ng/ml) + oxLDL (50 μg/ml) for 14 days, with immunostaining to alpha actin in cytoplasm of SMC, original magnification x40 **(A)**, and at x1,000 **(B)**. SMC foam cells were identified by Oil-red-O staining, original magnification x1000 **(C)**; and transmission electron microscope, original magnification x8,000 **(D)** from the same experiment comparing to those in the syngeneic HSV on day 0 at the same original magnification (inlets in **B**, **C**, and **D**). **(E)** Percentages of SMC foam cells in HSV rings were compared among all groups as mentioned above. Data represent means ± SEM from 3 - 6 independent experiments. Significant differences among groups are indicated; * p- valves = 0.00, and *** p- valves = not significant.

Some SMC in both TI and TM developed foam cells (SMC foam cells), which were identified by oil red O staining and TEM (Figure 3A-D). Figure 3E shows that the percentages of SMC foam cells within the total SMC in the vascular wall of T + nLDL (49.86 ± 2.43, p = 0.000), and T + oxLDL induced HSV rings (51.62 ± 1.72, p = 0.000) were significantly higher than that of the control (19.30 ± 1.71). No significant difference was found between T + nLDL and T + oxLDL stimulations (p = 0.999). Moreover, TNF-α alone (22.6 ± 5.11) had little effect on the number of SMC foam cells compared to the control (p = 0.993).

#### Increased expression of adhesion molecule, VCAM −1, in TI and TM

**Figure 4:**
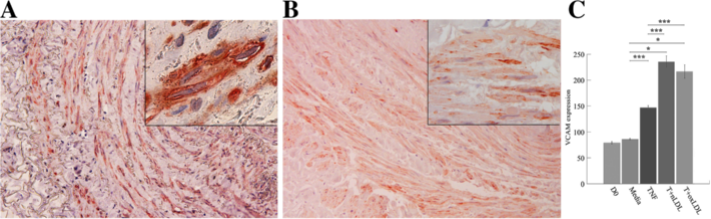
**T + LDL increased vascular adhesion molecule-1 (VCAM-1) expression in SMC of HSV cultures.** Double immunostaining of HSV rings cultured with T (5 ng/ml) + oxLDL (50 μg/ml) for 14 days, original magnification x 200 **(A)** compared to the syngeneic HSV on day 0 **(B)**; Each inlet shows the same picture at original magnification x 1,000. VCAM-1 and alpha actin positive reactivity was visualized with peroxidase substrate DAB (brown color) and Nova Red (red color), respectively. **(C)** Percentages of VCAM expressed SMC in HSV were compared among all groups as mentioned above. Data are means ± SEM from 3 - 6 independent experiments. Significant differences among groups are indicated; * p- valves = 0.00, and *** = not significant.

Expression of VCAM was mostly found in SMC in both TI and TM of HSV rings cultured with T + nLDL or T + oxLDL (Figure 4A). In Figure 4C, there is no significant difference in VCAM expression among HSV at D0 (79.0 ± 4.22), cultured in medium (86.2 ± 2.37, p = 1.000), and with TNF-α alone (146.67 ± 6.77, p = 0.455). In contrast, T + nLDL (235.5 ± 22.92, p = 0.000) and T + oxLDL (216.5 ± 26.0, p = 0.001) significantly induced VCAM-1 expression.

### Potential application of the model in anti-atherosclerotic agent study

E_2_ could modulate atherosclerotic plaque characteristics in HSV culture induced by T + nLDL or T + oxLDL

**Figure 5:**
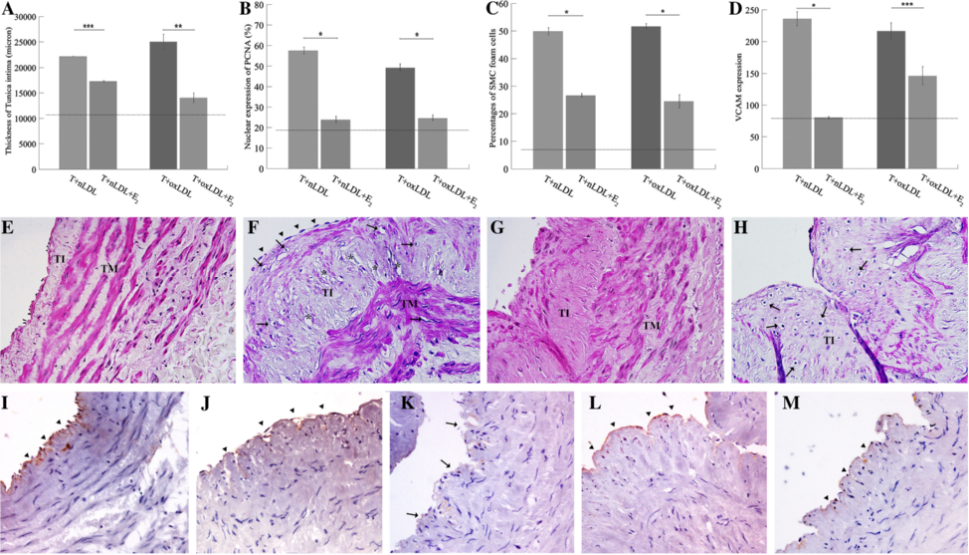
**E**_**2 **_**modulated atherosclerotic plaque characteristics in HSV culture induced by T + LDL.** The thickness of TI **(A)**, the percentage of PCNA expression **(B)** and foam cells **(C)** and the amount of VCAM expression **(D)** were modulated in the HSV rings cultured with T + nLDL + E_2_ (50 nM), or T + oxLDL + E_2_ (50 nM). Each dot line represents baseline valve of that in the syngeneic HSV rings on day 0. Significant differences among groups are indicated; * p - valves = 0.00, ** p - valves < 0.05, and *** = not significant. Photomicrographs illustrate histopathologic changes of the HSV rings cultured with T + oxLDL **(F)**, T + oxLDL + E_2_ (50 nM) **(G)**, or with T + oxLDL + E_2_ (25 nM) **(H)** for 14 days comparing to the syngeneic HSV on day 0 **(E)**. * indicates cholesterol cleft, arrow indicates foam cell, and arrow head points at the fibrous cap, TI = T. intima and TM = T. media (H&E staining, original magnification x 400). The endothelial linings of HSV rings at D0 **(I)**, cultured with TNF-α (5 ng/ml) **(J)**, T + oxLDL **(K)**, T + oxLDL + E_2_ (50 nM) **(L)**, or T + oxLDL + E_2_ (25 nM) **(M)** were localized by immunostaining with anti-vWf and peroxidase substrate DAB (original magnification x 400). Arrowheads and arrows point the positive and negative reactivity of EC, respectively.

Histological changes related to atherosclerotic plaque induced by T + nLDL and T + oxLDL induction were suppressed when 50 nM E_2_ was added in the HSV culture (Figure 5). The addition of E_2_ (25–50 nM) in the culture medium reduced the degree of injury of EC (Figure 5L, 5M).

Figure 5A-D reveal that E_2_ (50 nM) could decrease all parameters including the thickness of TI (17,297.5 ± 297.5 μm, p = 0.121), the number of PCNA positive SMC (23.8 ± 3.1%, p = 0.000), the number of SMC foam cells (26.60 ± 1.28%, p = 0.000), and the level of VCAM expression (80.25 ± 3.77, p = 0.000) in HSV rings cultured with T + nLDL + E_2_.

Similarly, 50 nM E_2_ in T + oxLDL could also reduce changes of the same set of parameters, including the thickness of TI (14,025.7 ± 1,945.02 μm, p = 0.034), the number of PCNA positive SMC (24.5 ± 2.7%, p = 0.001), the number of SMC foam cells (24.45 ± 4.83%, p = 0.000), and the level of VCAM expression (146.0 ± 28.74, p = 0.320).

Figure 5E-H are representative photomicrographs showing the effects of E_2_. Figure 5I-M represents results of immunostaining of vWf illustrating the EC integrity.

## Discussion

The present study is the first to use static HSV organ culture to study pathologic changes associated with atherogenesis by TNF-α in combination with nLDL or oxLDL. Following the theory of “Response to Injury”, we aimed to imitate the activation of vascular inflammation by hyperlipidemia in patients during the early stages of atherogenesis [1,5].

TNF-α is an important pro-inflammatory cytokine which 1) causes EC dysfunction by decreasing endothelial NO, which in turn up-regulates expression of adhesion molecules, increases proliferation and migration of SMC, and induces platelet aggregation [12], 2) induces SMC ScR expression and contributes SMC foam cell formation [15], and 3) enhances LDL oxidative modification by EC [17]. These alterations are consistent with the pathogenesis of the atherosclerosis as reviewed by Hopkins [26] and Hansson [27]. To study the role of TNF-α in our HSV model, we used recombinant human TNF-α, a strategy that allowed us to precisely control the concentration of TNF-α for initiating lesions. The dosage of TNF-α was adapted from previous publications [12,15,25].

Because TNF-α is not the only pro-inflammatory cytokine secreted from activated innate immunity cells, it will be important to develop a more sophisticated model which utilizes co-cultured innate immune cells. The complex interplay between cytokine signaling cells involved atherogenesis cannot be captured by our simple model.

nLDL or oxLDL in the culture medium mimicked hyperlipidemia in patients [5]. oxLDL in the culture passed through EC lining and TI via ScR on impaired EC [28]. Unlike oxLDL, nLDL required oxidation by TNF-α stimulated EC to become oxLDL [25,28] before entering TI. Foam cell formation is a hallmark of atherosclerotic plaques. Most foam cells arise from monocyte-derived macrophages recruited through EC stimulation by pro-inflamatory cytokines [19]. Foam cells develop when monocyte-derived macrophage or SMC within the arterial wall takes up oxLDL via ScR [27].

oxLDL (80 μg/ml) was found to be effective in enhancing vascular SMC migration within 24 hours and inducing foam cell formation in SMC in 72 hours [29]. In this study, addition of oxLDL (50 μg/ml) was sufficient to convert SMC in HSV to foam cell.

Some earlier studies used nLDL as a negative control for investigating the effects of oxLDL in EC-SMC culture [30,31]. nLDL itself could not induce foam cell formation because the receptor that binds to LDL is poorly expressed on differentiated macrophage. Moreover, the LDL receptor is not expressed in atherosclerotic plaques [32]. Our findings revealed however that T + nLDL can synergistically induced important features of atherosclerosis as effectively as T + oxLDL can.

TNF-α is predicted to cause EC dysfunction and decrease endothelial nitric oxide, leading to proliferation and migration of SMC in TI and TM. In addition, TNF-α is known to increase expression of ScR on SMC [25]. Uptake of oxLDL via ScR can lead to accumulation of cholesterol and develop SMC foam cells in both TI and TM. Therefore, although our HSV model does not directly incorporate monocytes, direct TNF-α stimulation can still increase the number of ScR on SMC and lead to significant SMC foam cell development useful for research.

In previous studies, Newby et al. studied the mechanism of atherosclerosis in HSV bypass in patients. They focused on spontaneous atherosclerosis development in HSV culture which imitates the conditions of HSV coronary bypass. This study undoubtedly had direct implications for coronary bypass patients [33]. Alternatively, Monaco et al. used atheroma cell mixtures from the human plaques lesions in short-term culture to study innate immunity in atherosclerosis. They investigated the roles of atheroma cells in human atherogenesis via TLR and MyD88 induced NF-kappa B activation [34]. Here, we used a different approach, aiming to generate atherosclerosis like lesions in normal HSV. By simultaneously adding LDL and TNF-α, which mimic the important risk factors of atherosclerogenesis: hyperlipidemia and inflammation, we can now reproduce several features of atherosclerosis in culture. Our model thus provides a novel tool for further atherogenesis and pharmacological studies.

Consistent with the previous studies [5,10,11,30], we found that activated SMC had important roles in the development of the thickness of stimulated HSV, which is dependent on SMC migration and proliferation [10,11], SMC foam cell accumulation [30], and expansion of extracellular matrix, and fibrous cap formation [5]. In our culture, proliferation of SMC was not due to implantation because we did not observe any attachment of SMC at the bottom of the culture plate, and the proliferation occurred specifically within TI and TM, which protruded into the lumen.

The Additional file 1 summarizes our unpublished data of an experiment of HSV stimulated with TNF-α and nLDL for 4 hours [35]. RNA was extracted and expression of atherosclerosis related genes was determined by Atherosclerosis quantitative RT-PCR array analysis (Qiagen, Hilden, Germany). 50 of 84 genes were up regulated (The Additional file 2). The activation was controlled by NF-kappa B1 (NFKB1), EGR1, TNF and TNFAIP3. Genes with markedly altered expressions included those encoded for lipid transport and metabolism, inflammation, adhesion molecules, extracellular molecules and cell proliferation (The Additional file 3). Our findings here are thus consistent with TNF-α and LDL could initiate the process of atherosclerosis and correlated to the phenotypic changes of our present model (Figures 1, 2, 3 and 4). Consistently, some studies have also suggested that NF-kappa B activation is induced by lipid [36] and TNF-α [37]. The down regulation of genes encoded for vWf supports our findings of injured EC integrity in HSV cultured with TNF-α + oxLDL (Figure 5K).

In the present study, we selected E_2_ to demonstrate an application of our initial plaque model. Previous studies have demonstrated beneficial effects of E_2_ as anti-atherogenesis in EC culture [12], animal models [8,38], and in human vein culture [39]. E_2_ stimulates nitric oxide production by promoting eNOS activity [12,39]. E_2_ also enhances endothelial integrity, EC proliferation, and survival. Furthermore, E_2_ can reduce permeability of EC to nLDL or oxLDL, inhibit SMC proliferation and migration, reduce adhesion molecule expression, and inhibit initiation of EC apoptosis induced by TNF-α and oxLDL (reviewed in [8]). Consistently, our phenotypic findings show that E_2_ (50 nM) also modulated the critical features of atherosclerotic plaque in our model, including decreased thickness of TI, proliferative index of SMC, SMC foam cell formation, VCAM-1 expression (Figure 5). Taken together, our model faithfully reports the pharmacological effects of E_2_.

### The limitations in this study

1) The size of this study is limited. Statistical bias might occur due to the small sample size. In addition, our study could not address the process and the alteration time course during the 14 day of culture. We could only demonstrate the terminal changes and compared among D0, and all conditions as mentioned above. 2) The immunological features should be evaluated further. We mainly investigated the phenotypic changes. We did not demonstrate monocyte adhesion and transmigration into TI. However, the marked increase of VCAM-1 expressed SMC in both TI and TM, which was in response to TNF-α induction, indirectly implied monocyte involvement in HSV culture. 3) Here, we aimed to utilize HSV segments, which were more easily obtained. We did not compare the changes of veins with those in artery cultures. However, a previous study [10] showed differences between vein and artery cultures. HSV develop significantly more intimal hyperplasia than the internal thoracic or radial artery cultures do in medium supplement with FBS (5-10%) and without any other stimulators. Therefore, we used D0 and cultures in medium alone as subtractive controls. In addition, Deng et al. [40,41] reported differences of gene expression of *in vitro* cultures of EC [40] and SMC [41] separated from arteries and veins after exposed to stimuli. TNF-α activated similar gene expression responses in both EC culture from coronary artery (CAEC) and HSV (SVEC). oxLDL induced greater gene expression in CAEC compared with SVEC [40], and stronger proliferative/migratory response in SMC culture from HSV [41]. Based on several previous findings [10,40,41], our findings are in agreement with expectations.

## Conclusions

Although mouse model is the most common used animal model and easy for genetic manipulation such as transgenesis, gene knock-out, and knock-in, its limitation is the lack of development of plaques with foam cell formation due to its different lipid profile compared to those in human. The present study was the first to apply TNF-α with LDL to enhance foam cell formation, and demonstrate SMC proliferation, SMC growth, and matrix accumulation within a short duration compared to animal models [5,7,8,42]. Therefore, our HSV culture model represents a novel tool for human atherosclerosis research.

## Abbreviations

TNF-α: Tumor necrosis factor-alpha; SMC: Smooth muscle cell; EC: Endothelial cell; HSV: Human saphenous vein; E2: 17-β estradiol; DAB: 3′ 3-diaminobenzidine; LDL: Low density lipoprotein; nLDL: native LDL; oxLDL: oxidized LDL; TI: Tunica intima; TM: Tunica media; TEM: Transmission electron microscopy; VCAM-1: Vascular cell adhesion molecule-1; ScR: Scavenger receptors; eNOS: endothelial nitric oxide synthase; PCNA: Proliferating cell nuclear antigen

## Competing interests

The authors declare that they have no competing interests.

## Authors’ contributions

KP and PC selected normal HSV segments. YM and KW were responsible for laboratory work including culture, and tissue staining. VK performed tissue preparation and processing. YM, UC and KW contributed to the histological, histophotometry and immunohistochemical examination. UC contributed to the ultrastructural study. SB carried out the real-time PCR assays and analyses. YM was responsible for data analysis and statistical calculation. YM conceived the study, and contributed to writing the manuscript. All authors interpreted the results, read and approved the final manuscript.

## Supplementary Material

Additional file 1 Determination of expression profile of atherosclerosis in the T+nLDL stimulated HSV segments. Click here for file

Additional file 2: Figure S1 Up-regulated expression of gene profiling of atherosclerosis (50 of 84 genes) performed by Atherosclerosis quantitative real time quantitative RT-PCR array analysis. (a) in a HSV segment cultured with T (5 ng/ml)+nLDL (50 μg/ml) as mentioned in Materials and Methods for 4 hours compared with the syngeneic segment cultured in medium alone (b). Numbers of genes are categorized into 8 functional groups (c). Click here for file

Additional file 3: Table S1 Up-regulated expression of gene profiling of atherosclerosis performed by Atherosclerosis quantitative real time quantitative RT-PCR array analysis. A HSV segment cultured with T (5 ng/ml)+nLDL (50 μg/ml) for 4 hours compared with the syngeneic segment cultured in medium alone. Click here for file
