# Supplementary material for: Phenotypic alterations in human saphenous vein culture induced by tumor necrosis factor-alpha and lipoproteins: a preliminary development of an initial atherosclerotic plaque model
Source: Lipids Health Dis. 2013 Sep 8;12:132. doi: 10.1186/1476-511X-12-132 (PMC3847608; doi:10.1186/1476-511X-12-132)
Supplement: Additional file 1 — Determination of expression profile of atherosclerosis in the T+nLDL stimulated HSV segments. [file 1476-511X-12-132-S1.doc]

**The Additional file 1:**

**Determination of expression profile of atherosclerosis in the T+nLDL stimulated HSV segments**

**Methods:**

**Experimental design**

Segments of HSV were cultured in DMEM supplement with 5% of FBS, T (5ng/ml) + nLDL (50 μg/ml). After 4 hours of cultivation^31^, total RNA was extracted from the stimulated HSV segment and in medium alone. The atherosclerosis related mRNA expression was investigated using the Human Atherosclerosis RT^2^ Profiler^TM^ PCR Array. The up-regulated expression profile was analyzed in comparison to that of the syngeneic vein cultured in medium alone by Light Cycler ® 480 software, SABiosciences, QIAGEN, Germany).

***Quantitative real time RT-PCR microarray analysis***

Quantitative gene expression profile of atherosclerosis analysis was performed using kit according to the instruction from the manufacturer. Briefly, total RNA in the cultured HSV rings were preserved in 1 ml of Trizol and kept at -70^o^C until extraction. Then, RNA was extracted according to the standard method. The extracted RNA was further purified and performed DNA clean up by Qiagen RNAeasy kit, then determined the purity and quality by Nanodrop Spectrophotometry. The total RNA was further reverse transcripted to cDNA.

***Quantitative real time PCR array***

Quantitative real time PCR was performed using the Human Atherosclerosis RT^2^ Profiler^TM^ PCR Array. The up-regulated expression profile was analyzed in comparison to that of the syngeneic vein cultured in medium alone by Light Cycler® 480 software, SABiosciences, QIAGEN, Germany). Samples were run in 96 well plates. PCR cycling condition were 95^o^C for 5 minutes, then 45 cycles of 95^o^C for 15 seconds, and 60^o^C for 1 minute. The atherosclerosis array contained RT^2^ qPCR Primer assays for a set of 84 related genes, and 5 housekeeping genes. The RT control, positive PCR control and human genomic DNA contamination were included to quarantine the quality of PCR products.

For PCR array analysis, the up and down regulated gene expressions were determined at 2 fold changes in comparison to the syngeneic HSV cultured in medium alone.

**Results and Discussion:**

***Expression profiles of atherosclerosis in HSV stimulated with T+nLDL for 4 hours***

About 50 of 84 genes of atherogenesis were up regulated expressed as shown in the **Addtional file 2 (Figure S1) and 3 (Table S1)**. The activation was mainly regulated via transcription regulators mainly NF-kappa B1 (NFKB1) and minor via EGR1, TNF and TNFAIP3. The marked gene expressions included gene groups encoded for lipid transport and metabolism, inflammation, adhesion molecules, extracellular molecules and cell proliferation. These findings of gene profiling were consistent with the phenotypic criteria features of atherosclerotic plaques like lesion in our 14-day HSV ring culture.

**The Additiaonal file 2 Description:**

**Up-regulated expression of gene profiling of atherosclerosis (50 of 84 genes) performed by Atherosclerosis quantitative real time quantitative RT-PCR array analysis**.

(a) in a HSV segment cultured with T (5 ng/ml)+nLDL (50 μg/ml) as mentioned in Materials and Methods for 4 hours compared with the syngeneic segment cultured in medium alone (b). Numbers of genes are categorized into 8 functional groups (c).

**The Additional file 3 Description:**

**Up-regulated expression of gene profiling of atherosclerosis performed by Atherosclerosis quantitative real time quantitative RT-PCR array analysis.** A HSV segment cultured with T (5 ng/ml)+nLDL (50 μg/ml) for 4 hours compared with the syngeneic segment cultured in medium alone.
