# Supplementary figures and images for: Phenotypic alterations in human saphenous vein culture induced by tumor necrosis factor-alpha and lipoproteins: a preliminary development of an initial atherosclerotic plaque model
Source: Lipids Health Dis. 2013 Sep 8;12:132. doi: 10.1186/1476-511X-12-132 (PMC3847608; doi:10.1186/1476-511X-12-132)

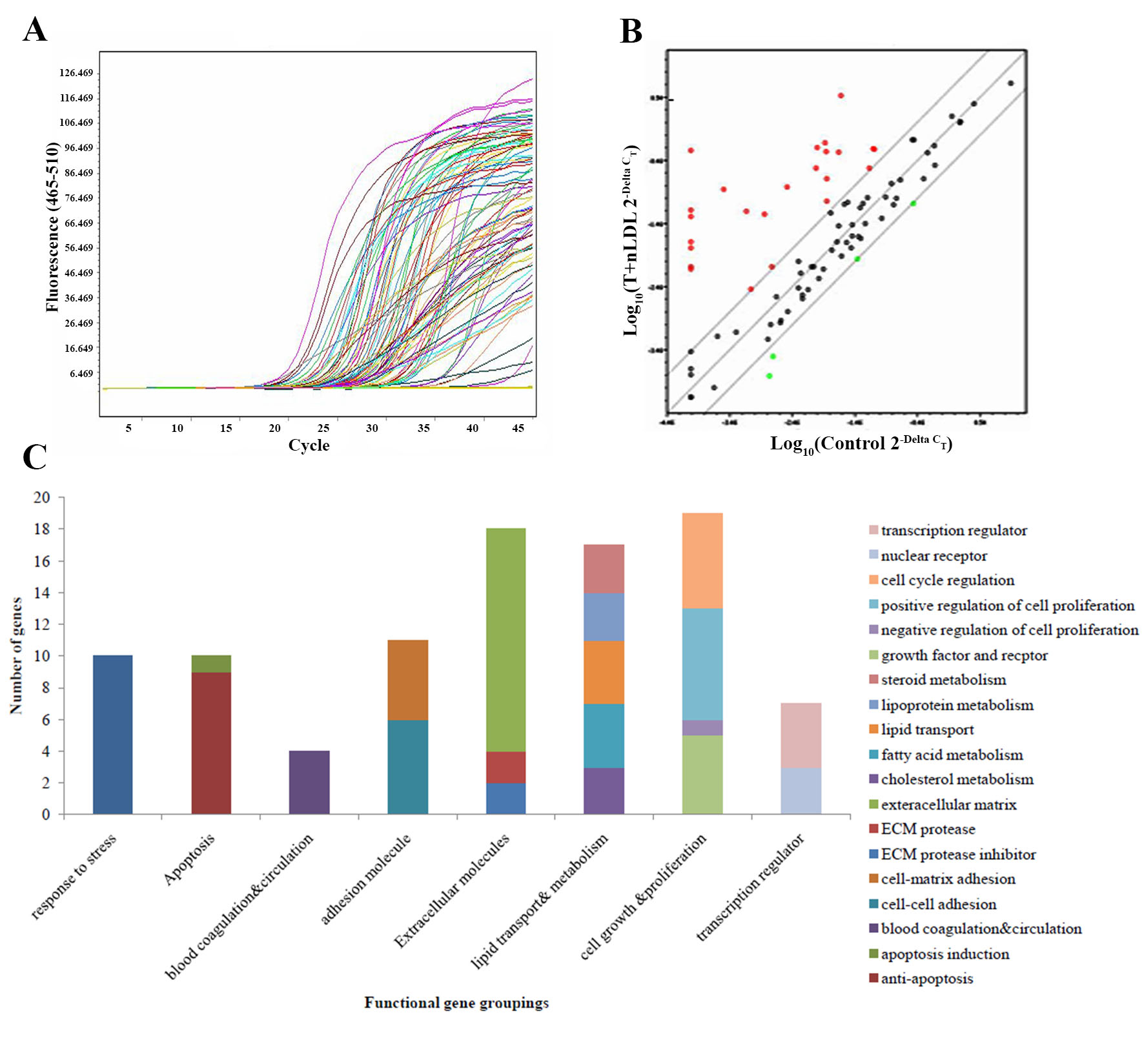

Supplement: Additional file 2: Figure S1 — Up-regulated expression of gene profiling of atherosclerosis (50 of 84 genes) performed by Atherosclerosis quantitative real time quantitative RT-PCR array analysis. (a) in a HSV segment cultured with T (5 ng/ml)+nLDL (50 μg/ml) as mentioned in Materials and Methods for 4 hours compared with the syngeneic segment cultured in medium alone (b). Numbers of genes are categorized into 8 functional groups (c). [file 1476-511X-12-132-S2.jpeg]
