# Supplementary material for: Phenotypic alterations in human saphenous vein culture induced by tumor necrosis factor-alpha and lipoproteins: a preliminary development of an initial atherosclerotic plaque model
Source: Lipids Health Dis. 2013 Sep 8;12:132. doi: 10.1186/1476-511X-12-132 (PMC3847608; doi:10.1186/1476-511X-12-132)
Supplement: Additional file 3: Table S1 — Up-regulated expression of gene profiling of atherosclerosis performed by Atherosclerosis quantitative real time quantitative RT-PCR array analysis. A HSV segment cultured with T (5 ng/ml)+nLDL (50 μg/ml) for 4 hours compared with the syngeneic segment cultured in medium alone. [file 1476-511X-12-132-S3.pdf]

Table 1 Expression profiles of atherosclerosis in HSV stimulated with T+nLDL for 4 hours

Gene symbol	Description	2 fold changes	Functional gene groupings							
			Response to stress	Apoptosis	Coagulation & Circulation	Adhesion molecules	Extracellular molecules	Lipid transport & metabolism	Cell growth & proliferation	Transcriptio regulation
ABCA1	ATP-binding cassette, sub-family A (ABC1), member 1	3.5308								
ADFP	Adipose differentiation-related protein	7.2602						✓		
APOA1	Apolipoprotein A-I	2.5847						✓		
BCL2A1	BCL2-related protein A1	85.6274		✓						
BID	BH3 interacting domain death agonist	1.021		✓						
BIRC3	Baculoviral IAP repeat-containing 3	14.2215		✓						
CCL2	Chemokine (C-C motif) ligand 2	174.8532	✓	✓		✓	✓			
CD44	CD44 molecule (Indian blood group)	2.5669								
CSF1	Colony stimulating factor 1 (macrophage)	1.5476								
CSF2	Colony stimulating factor 2 (granulocyte-macrophage)	3.8906					✓		✓	
EGR1	Early growth response 1	39.3966								

Gene symbol	Description	2 fold changes	Functional gene groupings							
			Response to stress	Apoptosis	Coagulation & circulation	Adhesion molecules	Extracellular molecules	Lipid transport & metabolism	Cell growth & proliferation	Transcriptio regulation
**FGF2**	**Fibroblast growth factor 2 (basic)**	**1.6021**							✓	
**HBEGF**	**Heparin-binding EGF-like growth factor**	**45.8866**							✓	
**ICAM1**	**Intercellular adhesion molecule 1 (CD54), human rhinovirus receptor**	**64.4452**				✓				
**IFNAR2**	**Interferon (alpha, beta and omega) receptor 2**	**1.2142**								
**IFNG**	**Interferon, gamma**	**171.2547**								
**IL1A**	**Interleukin 1, alpha**	**210.8393**							✓	
**IL1R1**	**Interleukin 1 receptor, type I**	**4.5002**								
**IL1R2**	**Interleukin 1 receptor, type II**	**4.2281**	✓							
**ITGA5**	**Integrin, alpha 5 (fibronectin receptor, alpha polypeptide)**	**2.4116**				✓				
**ITGAX**	**Integrin, alpha X (complement component 3 receptor 4 subunit)**	**2.0994**				✓				
**KDR**	**Kinase insert domain receptor (a type III receptor tyrosine kinase)**	**1**							✓	

Gene symbol	Description	2 fold changes	Functional gene groupings							
			Response to stress	Apoptosis	Coagulation & circulation	Adhesion molecules	Extracellular molecules	Lipid transport & metabolism	Cell growth & proliferation	Transcription regulation
**LDLR**	**Low density lipoprotein receptor (familial hypercholesterolemia)**	**24.2515**						✓		
**LIF**	**Leukemia inhibitory factor (cholinergic differentiation factor)**	**436.5491**					✓			
**MMP1**	**Matrix metallopeptidase 1 (interstitial collagenase)**	**680.2871**					✓			
**MMP3**	**Matrix metallopeptidase 3 (stromelysin 1, progelatinase)**	**39.1245**					✓			
**MSR1**	**Macrophage scavenger receptor 1**	**2**								
**NFKB1**	**Nuclear factor of kappa light polypeptide gene enhancer in B-cells**	**5914.334**	✓	✓						✓
**NOS3**	**Nitric oxide synthase 3 (endothelial cell)**	**4.4691**	✓							
**NPY**	**Neuropeptide Y**	**1.6818**							✓	
**PDGFA**	**Platelet-derived growth factor alpha polypeptide**	**1.6472**			✓					
**PPARD**	**Peroxisome proliferator-activated receptor delta**	**2.9485**								✓
**PPARG**	**Peroxisome proliferator-activated receptor gamma**	**1.2397**								✓

Gene symbol	Description	2 fold changes	Functional gene groupings							
			Response to stress	Apoptosis	Coagulation & circulation	Adhesion molecules	Extracellular molecules	Lipid transport & metabolism	Cell growth & proliferation	Transcription regulation
**RXRA**	**Retinoid X receptor, alpha**	**2.8481**								✓
**SELE**	**Selectin E (endothelial adhesion molecule 1)**	**533.7425**	✓							
**SERPINB2**	**Serpin peptidase inhibitor, clade B (ovalbumin), member 2**	**79.3413**					✓			
**SERPINE1**	**Serpin peptidase inhibitor**	**7.7812**			✓		✓			
**SPP1**	**Secreted phosphoprotein 1**	**1.5052**								
**TGFB1**	**Transforming growth factor, beta 1**	**1.057**							✓	
**TGFB2**	**Transforming growth factor, beta 2**	**1.1329**							✓	
**TNF**	**Tumor necrosis factor (TNF superfamily, member 2)**	**84.4485**	✓			✓				✓
**TNFAIP3**	**Tumor necrosis factor, alpha-induced protein 3**	**57.2816**								
**VCAM1**	**Vascular cell adhesion molecule 1**	**6.3203**				✓				
**VEGFA**	**Vascular endothelial growth factor A**	**31.5594**				✓				
**B2M**	**Beta-2-microglobulin (house keeping gene)**	**1.3947**								

Gene symbol	Description	2 fold changes	Functional gene groupings							
			Response to stress	Apoptosis	Coagulation & circulation	Adhesion molecules	Extracellular molecules	Lipid transport & metabolism	Cell growth & proliferation	Transcriptio regulation
**HPRT1**	**Hypoxanthine phosphoribosyltransferase 1 (house keeping gene)**	**1.4641**								
**RTC**	**Reverse Transcription Control**	**2.9897**								
**RTC**	**Reverse Transcription Control**	**1.2924**								
**PPC**	**Positive PCR Control**	**2.2346**								
**PPC**	**Positive PCR Control**	**1.3195**								
**PPC**	**Positive PCR Control**	**9.9177**								
